# Cohort Study of Risk Factors for Breast Cancer in Post Menopausal Women

**DOI:** 10.4178/epih/e2013003

**Published:** 2013-04-30

**Authors:** Arthur J. Hartz, Tao He

**Affiliations:** Health Services Research, Huntsman Cancer Institute, University of Utah School of Medicine, Salt Lake City, UT, USA.

**Keywords:** Breast cancer, Risk factors, Obesity, Postmenopausal hormone replacement therapy, Thyroid cancer, Breast augmentation

## Abstract

**OBJECTIVES:**

The present study assessed more than 800 potential risk factors to identify new predictors of breast cancer and compare the independence and relative importance of established risk factors.

**METHODS:**

Data were collected by the Women's Health Initiative and included 147,202 women ages 50 to 79 who were enrolled from 1993 to 1998 and followed for 8 years. Analyses performed in 2011 and 2012 used the Cox proportional hazard regression to test the association between more than 800 baseline risk factors and incident breast cancer.

**RESULTS:**

Baseline factors independently associated with subsequent breast cancer at the p<0.001 level (in decreasing order of statistical significance) were breast aspiration, family history, age, weight, history of breast biopsies, estrogen and progestin use, fewer live births, greater age at menopause, history of thyroid cancer, breast tenderness, digitalis use, alcohol intake, white race, not restless, no vaginal dryness, relative with prostate cancer, colon polyps, smoking, no breast augmentation, and no osteoporosis. Risk factors previously reported that were not independently associated with breast cancer in the present study included socioeconomic status, months of breast feeding, age at first birth, adiposity measures, adult weight gain, timing of initiation of hormone therapy, and several dietary, psychological, and exercise variables. Family history was not found to alter the risk associated with other factors.

**CONCLUSIONS:**

These results suggest that some risk factors not commonly studied may be important for breast cancer and some frequently cited risk factors may be relatively unimportant or secondary.

## INTRODUCTION

Breast cancer risk factors have been studied often. The present study has the advantage of simultaneously considering many risk factors. This makes it possible to find new risk factors, assess the relative importance of risk factors, and assess which risk factors are secondary (their association is removed by adjusting for other risk factors). The data for the present study were collected by the Women's Health Initiative (WHI). Because the WHI is a prospective study with a large, comprehensive, meticulously collected database, it is especially well-suited for evaluating risk factors.

## MATERIALS AND METHODS

The WHI study design has been described in detail [1]. In brief, it was a long-term national health study that focused on strategies for preventing heart disease, breast and colorectal cancer, and osteoporosis in postmenopausal women. Women between the ages of 50 and 79 were enrolled in either an observational study (OS) or a randomized controlled trial (RCT) from 1993 to 1998 at 40 clinical centers throughout the United States and followed for a median time of 8 years to ascertain outcomes. The study was approved by institutional review boards, and all participants signed informed consent forms.

Participants available for analysis included 161,807 WHI participants: 93,675 from the OS, 16,608 from the RCT of estrogen plus progesterone (E+P), 10,739 from the RCT of estrogen only (estrogen-alone), and 40,785 additional women who were in the diet study and not in a RCT of hormone therapy. To minimize the inclusion of participants with a history of breast cancer, we excluded from the present analysis those who reported a history of breast cancer, a breast removed, or current antineoplastic therapy. Of the 161,807 participants in the dataset we excluded 7,906 because of a possible history of breast cancer and 825 patients who could not influence the analysis because they did not have follow-up. An additional 5,874 participants were excluded from the final analyses because they did not have data on all factors independently associated at the p<0.001 level with the development of breast cancer. There were 147,202 women included in the final analyses.

### Data

Several data sources were used to determine breast cancer recurrence and the date of this recurrence [1]. For follow-up and outcome ascertainment all subjects completed a self-administered, self-report. This report was completed semiannually by the RCT participants and annually by the OS participants. Medical records were reviewed for patients who died in the hospital, and autopsy reports were reviewed for patients who had an autopsy. Only the death certificates were reviewed for patients who died outside of the hospital without an autopsy.

There were 869 factors that characterized subjects at baseline that were evaluated for an association with the risk of breast cancer for all participants and an additional 102 factors were evaluated for an association with participants in the OS. Types of information included demographic, general health, clinical and anthropometric, functional status, healthcare behaviors, reproductive, medical history, family history, personal habits, thoughts and feelings, therapeutic class of medication, hormones, supplements, and dietary intake. Scales used in this study that we anticipated might be important included stressful life events (a scale from 0 to 33 from the Alameda County Study), optimism (a 6 to 30 scale from the Life Orientation Test-Revised) and depression (a 0 to 1 scale from the Center for Epidemiological Studies).

### Statistical methods

Analyses were performed in 2011 and 2012 using the Cox proportional hazard regression model to find the hazard ratio of breast cancer associated with a factor after adjusting for other risk factors. We first tested the statistical significance of each potential risk factor after adjusting for only the study that recruited the patient, age, and race. All variables that were statistically significant at the p<0.05 level were then included in a backwards stepwise Cox proportional hazard regression analysis, and variables that were statistically significant at the p<0.001 level were retained in the model. We then retested the statistical significance of all variables not in the model to determine if any should be added because they would be significant at the p<0.001 level. The significance level of the variables was not adjusted for multiple comparisons because this might obscure meaningful results.

The hazard ratio was usually reported for a one unit increase in the risk factor. For age risk was measured for a 10 year increase, and where noted the hazard ratio was for an increase in one standard deviation of the risk factor. For categorical variables a reference category was chosen, and the hazard ratios were shown for other categories compared to the reference category. Some ordinal variables were also divided into categories, and in addition to an overall hazard ratio and significance test for the ordinal variable there was a hazard ratio comparing a given category to the reference category.

To test whether the risks associated with some variables were influenced by others, we tested pairs of the statistically significant independent variables for interaction.

In addition to testing the variables in the complete dataset, we also tested the results for participants in each of three subsets: 1) the OS, 2) the RCT of diet, and 3) the combination of the two RCTs of hormone therapy that were each relatively small.

Missing data for variables that were not independently significant were imputed prior to the analysis of these variables by the mean value for ordinal or binary variables and the mode value for categorical variables.

Statistical analyses were performed using SAS version 9.0 (SAS Inc., Cary, NC, USA).

## RESULTS

The demographic characteristics of the participants are shown in Table 1. Most were white, between the ages of 55 and 70, and with more than a high school education. The regions of the United States were equally represented.

### Demographic factors

As shown in Table 2 three demographic factors (age, white race, and specific study to which subjects were enrolled) have an independent statistically significant association (p<0.001) with breast cancer after adjusting for all factors independently significant at the p<0.001 level. The association of age and race with breast cancer did not vary significantly across the three datasets. Other variables in Table 2 (college education, income, professional or managerial occupation, working with hair dyes for one year) had an association with breast cancer at the p<0.0001 level when only adjusting for age, race, and specific study. However, the significance was reduced after adjusting for the other variables that were statistically significant at the p<0.001 level. Working with hair dyes seemed to be associated with breast cancer in a univariate analysis only because it was a marker for socioeconomic status, 3.5% of 34,234 women with family incomes of less than $50,000 reported working with hair dyes as compared to 1.2% of the 35,094 women with family incomes of $50,000 or more.

### Breast and reproductive factors

The highest chi-squared values in Table 3 were for the number of needle aspirations of breast cysts, having a first degree relative with breast cancer, and having a biopsy of lesions suspicious of breast carcinoma. A history of hormone therapy was also important. The number of years of using hormone therapy and whether or not hormone therapy was used at baseline were independent risk factors. The risk of breast cancer was 55% greater for women on estrogen and progesterone for more than 15 years compared with women not on hormones. The hazard ratio for women taking estrogen plus progesterone at baseline, 1.29, was greater than the hazard ratio for women taking estrogen alone, 1.14.

The best way to show how hazard ratios for continuous variables varied across data sets was to compare the hazard ratio for the continuous form of variable. The confidence intervals for categories of continuous variables in the individual datasets were wide and comparisons were less meaningful. The number of years of hormone use had a stronger association with subsequent breast cancer in the observational and RCT for diet datasets than it did in the RCTs for hormone therapy in these RCTs the past use of hormones had no association with the future use, which was determined by random assignment. The hazard ratios were similar for the observational study and the RCT for baseline use of estrogen plus progesterone but not for baseline use of estrogen alone.

The risk of breast cancer decreased for women as they had more children, up to a 39% reduction for women who had seven or more births. Later age at menopause, breast tenderness and benign breast disease were associated with a higher risk, while vaginal dryness and breast augmentation were associated with lower risk.

Participants who had a mammogram were at higher risk for breast cancer although this was greatly reduced after taking into account the variables that were independently significant. There may have been a protective effect of having an oophorectomy prior to age 40. Neither first birth after age 30 nor breast feeding for at least 2 years had a statistically significant independent association with breast cancer. Later age at menarche had a protective but weak association with breast cancer.

### Health and health behaviors

As shown in Table 4 the factor in this group most significantly associated with breast cancer was weight (chi-squared=93.8). The chi-squared value for weight was higher than for waist measurement, body mass index, or waist-hip ratio. After adjusting for weight, the chi-squared values for these variables were not statistically significant. It was only possible to calculate change in weight (i.e., the difference between current weight and minimum adult weight) in the observational study. Change in weight was not significant after adjusting for current weight although it was highly significant prior to this adjustment.

The risk of breast cancer also increased with history of thyroid cancer, the use of cardiotonic medications (which additional review of the data showed was almost always digitalis), greater alcohol consumption, a history of colon polyps, greater smoking, and a relative with prostate cancer. It decreased for women who reported themselves to be restless and fidgety or having a history of osteoporosis. Although the hazard ratios for some risk factors (e.g., thyroid cancer and relatives with prostate cancer) varied considerably among the datasets, this variation was not statistically significant.

### Nonsignificant factors hypothesized to influence risk

Factors previously evaluated in the literature that were not associated with breast cancer at the p<0.001 level after adjusting for age, race, and study are shown in Table 5. The factors related to psychological well-being and diet were not found to be associated with breast cancer. Other dietary factors that were not statistically significant and not included in the table were sugar, carbohydrates, glycemic index, protein, vegetables, dietary fiber, and caffeine. The only variable in the table that was statistically significant at even the p<0.05 level was the metabolic equivalents of walking times the number of hours of walking per week. This exercise variable included in the table was the most statistically significant of a total of the 23 exercise measures collected by WHI.

Not shown in Table 5 are tests of whether the associations of some factors with breast cancer were influenced by other risk factors. We did not find evidence even at the p<0.05 level that the association of breast cancer with number of breast aspirations, number of live births, or alcohol consumption were significantly influenced by family history of breast cancer or age. There was also no evidence that the risk of breast cancer associated with estrogen plus progesterone increased as weight increased.

## DISCUSSION

A large, diverse study population with long follow-up and comprehensive participant information was used to evaluate numerous potential risk factors for breast cancer in postmenopausal women. The study found many factors associated with breast cancer and 20 independently associated at the p<0.001 level of statistical significance. The large sample size made it possible to detect relatively weak associations and to precisely estimate the strength of association between the risk factor and breast cancer. Because numerous factors were examined, it was possible to show that some factors mediated the risk associated with others. Associations between a risk factor and outcome disappeared after adjusting for mediating risk factors.

The statistically significant independent risk factors in decreasing order of their chi-squared values for one degree of freedom were number of previous breast aspirations, breast cancer in first degree relatives, age, weight, a previous breast biopsy, years using estrogen and progesterone, fewer live births, use of estrogen and progesterone at baseline, age at menopause, a history of thyroid cancer, breast tenderness, digitalis use, at least one drink per week, less restlessness, lack of vaginal dryness, relative with prostate cancer, colon polyps, no breast augmentation, smoking more than 15 cigarettes a day, and no osteoporosis.

Factors of little importance after adjusting for the independent risk factors included socioeconomic status, breast feeding, age at first birth, obesity measures, adult weight gain after adjusting for current weight, and several dietary, psychological, and exercise variables. There was no evidence that family history influenced the association of breast cancer with the number of live births or alcohol consumption. There was also no evidence that greater weight reduced the risk associated with estrogen plus progesterone.

Greater weight may be associated with breast cancer because it is associated with hormone levels likely to contribute to breast cancer including higher leptin levels, lower adiponectin levels, and higher estrogen levels [2]. Weight is also associated with height, which is a risk factor [3]. The association of a biopsy with breast cancer is thought to be because of the condition that prompted the biopsy [4]. The number of breast aspirations may be an important risk factor because women who had multiple aspirates may have had multiple large cysts, a condition which has been associated with an increased risk of breast cancer for women with a family history of breast cancer [5]. However, we did not find an increase in the association between breast cancer and aspirations for women who had a family history of breast cancer. This increase would be expected if multiple cysts were only a risk factor for women with a family history of breast cancer.

Some variables highly associated with breast cancer in this study directly increase exogenous hormone levels. Others such as number of births and age at menopause indirectly influence hormone levels by influencing the number of lifetime menstrual cycles [6]. The risk associated with digitalis may be because of its estrogenic effect [7], and the protective effect of vaginal dryness and osteoporosis may be because these conditions are markers for relatively low estrogen levels. Even the protective association of restlessness may be because it indicates lower levels of estrogen although this is not well established [8,9]. Breast augmentation may have a protective association because it is a marker for a smaller breast size, which some studies have suggested is associated with reduced risk [10,11]. In any case there is no evidence from this study that breast implants increase the risk of cancer.

One of the highest hazard ratios was seen with thyroid cancer. This might have been secondary to the radiation exposure in the treatment for thyroid cancer. It is also possible that both the thyroid cancer and the breast cancer may have been increased by medical radiation as treatment for benign conditions such as acne or for medical or dental diagnostic tests that used excessive radiation [12].

### Previous epidemiologic studies

A well established set of risk factors commonly used in studies of breast cancer [13] include age at menarche, age at first live birth, number of previous biopsies, and number of first-degree relatives with breast cancer. These risk factors were also found to be statistically significant in the present study although age at menarche was much less significant than others, and age at first birth was not independently significant. Other previously identified risk factors [14] found important in the present study include age, age at menopause, weight, alcohol intake (at least in some studies [15] although not others [16]) hormone therapy, especially estrogen-progestin combinations,and higher bone mineral density [17]. Statistically significant but less important risk factors that had been identified previously include white race [18] and having a male relative with prostate cancer [19]; the latter risk factor has not always been significant when tested [20]. A study using National Cancer Institute's Surveillance, Epidemiology, and End Results data found a much lower risk of breast cancer associated with thyroid cancer, 1.21 [21], than in the present study Another study found that women who had been treated with radioactive iodine had a rate of breast cancer that was 90% greater than in the general population although this increase did not reach statistical significance at the p<0.05 level [22].

Some risk factors found in previous literature were weaker in this study (e.g., exercise [23]) some were not statistically significant (e.g., dietary calories and fat [24], dietary meat [25], and birth weight [26]), and others are entirely explained by the statistically independent risk factors in this study (e.g., adult weight gain [27-30], greater waist girth [28], and socioeconomic status [31,32]). It is possible that the weak association with breast cancer for some risk factors in this study (e.g., age at menarche) was because the variable was inaccurately reported by the participants. We did not find evidence that stressful life events were risk factors, which agrees with some publications [33] but not others [34]. Our results also did not support previous work showing the influence of a family history of breast cancer on the association between breast cancer and number of children [35] or alcohol [36].

We found evidence of a weak association between breast cancer and smoking that had been found in other studies [37] although in contrast to a previous study [37] we did not find that beginning smoking during the teenage years was an independent risk factor. As with other studies there was no association between breast cancer and diet (including total calories, calories from fat, or folic acid) [38]. Our findings on increased risk associated with breast tenderness and decreased risk associated with hair dyes were opposite of what has been found previously [39,40].

We are unaware of studies that evaluated some risk factors that were independently significant in this study: number of breast aspirations, vaginal dryness, breast augmentation, and restlessness.

This study assessed the relative importance of several risk factors for breast cancer and identified some new risk factors. Some previously identified factors were only weakly associated with breast cancer after adjusting for other risk factors.

## Figures and Tables

**Table 1:**
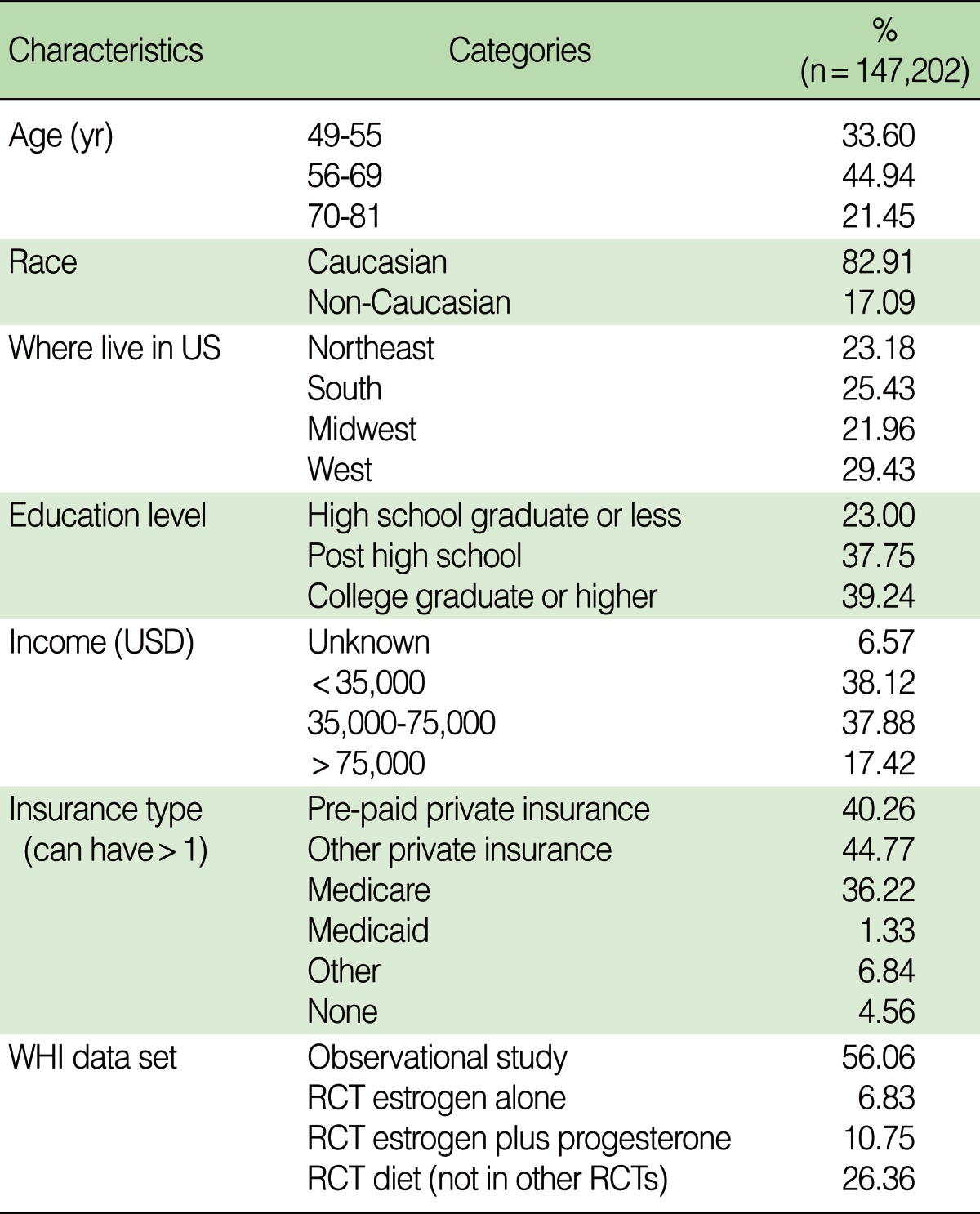
Demographic characteristics

USD, United States dollar; WHI, Women's Health Initiative; RCT, randomized controlled trial.

**Table 2:**
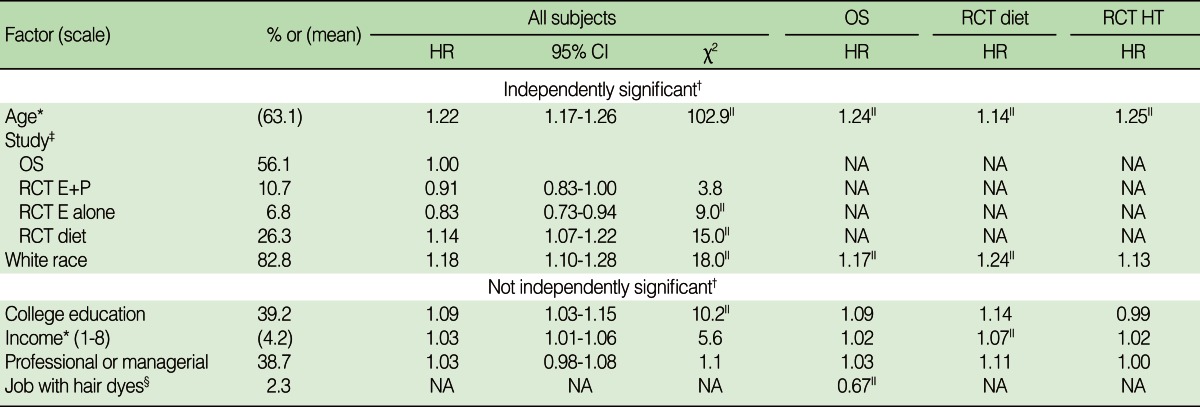
The association of demographic factors with breast cancer

HR, hazard ratio; CI, confidence interval; OS, observational study; RCT, randomized controlled trial; RCT HT, combination of the RCTs for E+P and for E-alone; E+P, estrogen plus progesterone; E-alone, estrogen alone. ^*^The hazard ratio was computed for an increase in the variable of one standard deviation; ^†^A variable is independently significant if p<0.001 after adjusting for all other independently significant variables; ^‡^Hazard ratios for a given study compare the breast cancer risk for participants in that study to the participants in the observational study; ^§^Subjects who worked with hair dyes for >1 year were compared to all other subjects; ^∥^The hazard ratio is statistically significant at p<0.01.

**Table 3:**
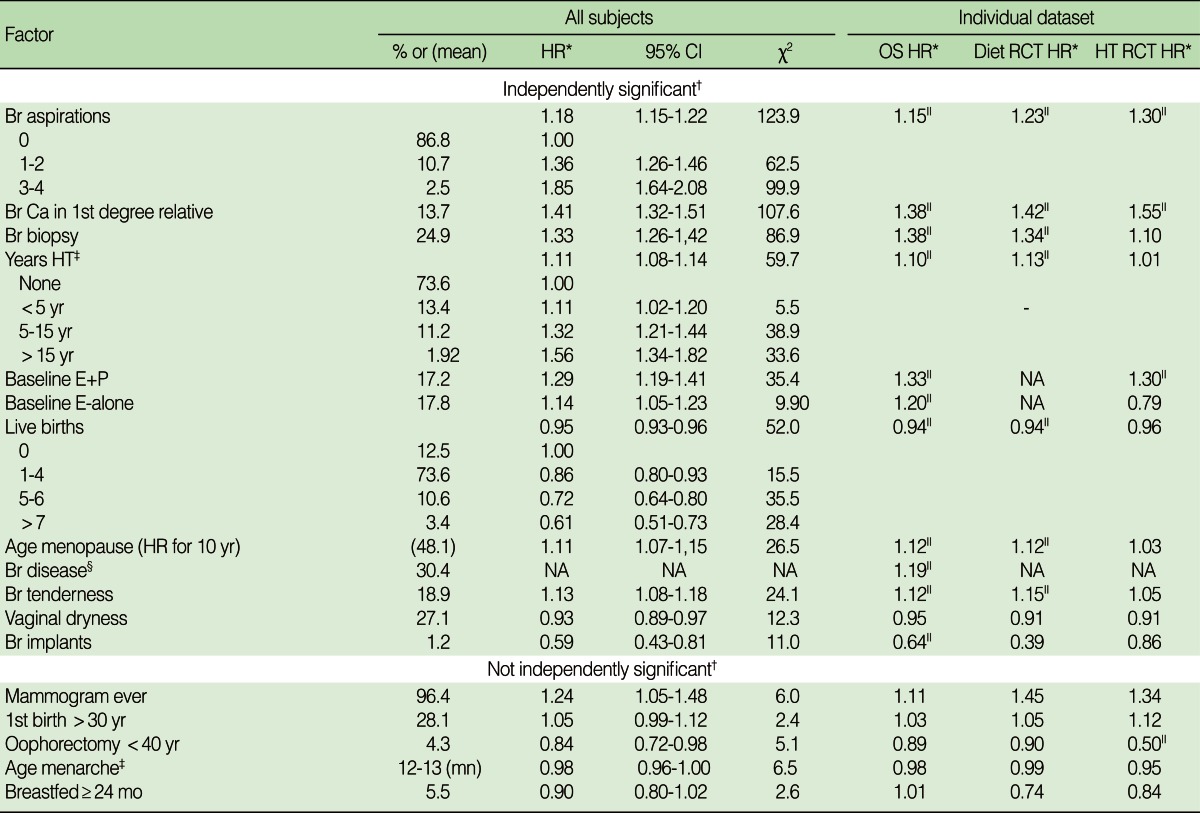
The association^*^ of familial, breast, and reproductive factors with breast cancer

Br, breast; CI, confidence interval; E+P, estrogen plus progesterone at baseline; E only, estrogen alone at baseline; HR, hazard ratio; HT, either E+P or E-alone; Mn, mean; OS, observational study; RCT, randomized controlled trial. ^*^Hazard ratios were computed for an increase of 1 unit of the ordinal variable or comparison to the reference category of that variable. This reference category has a hazard ratio of 1.00. Hazard ratios were only compared for ordinal variables; other comparisons would be based on less precise estimates; ^†^Factors are labeled as independently significant if their p-value <0.001 after adjusting for all other independently significant variables. Listed factors that were not independently significant had a p value of <0.001 after adjusting for age, race and study. Their tabulated chi-squared value were obtained after adjusting for the independently significant variables; ^‡^The hazard ratio was computed for an increase in the variable of one standard deviation; ^§^The doctor said there was benign breast disease. This information was only collected for patients in the observational study. The hazard ratio for benign breast disease was adjusted for all other variables with p<0.001 including breast aspiration and breast biopsy; ^∥^The hazard ratio is statistically significant at p<0.01 in this dataset.

**Table 4:**
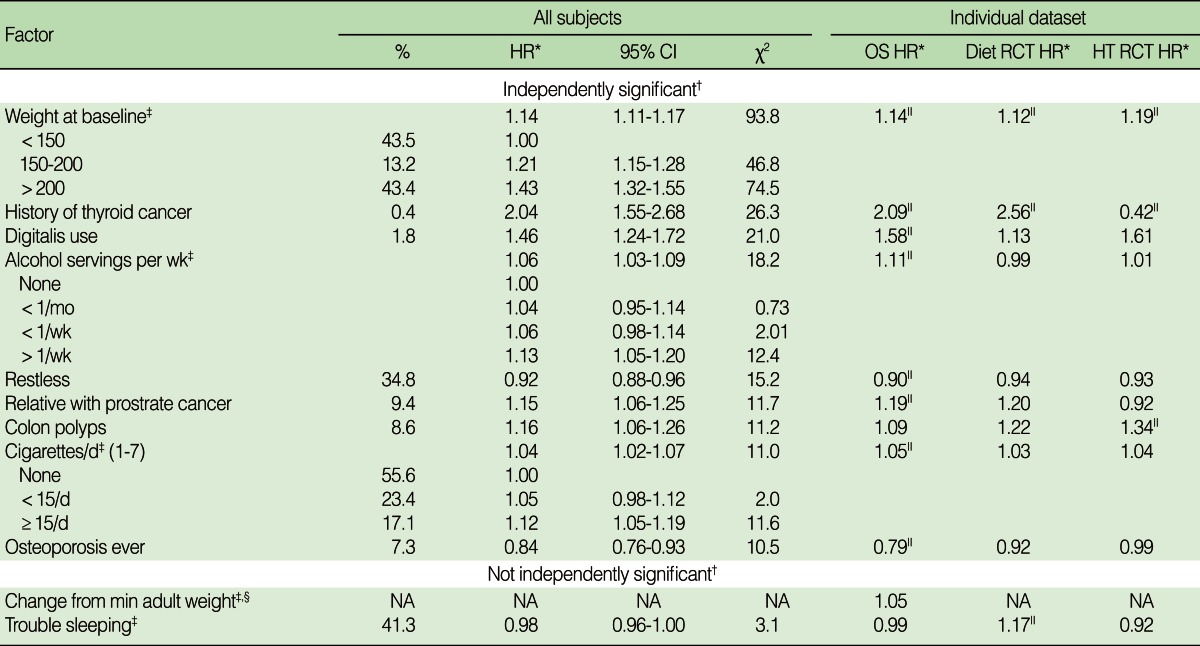
The association^*^ of health and behaviors with the development of breast cancer

HR, hazard ratio; CI, confidence interval; OS, observational study; RCT, randomized controlled trial; RCT HT, a combination of two RCTs; Min, minimum. ^*^Hazard ratios were computed for an increase of 1 unit of the ordinal variable or comparison to the reference category of that variable. This reference category has a hazard ratio of 1.00. Hazard ratios were only compared for ordinal variables; other comparisons would be based on less precise estimates; ^†^Factors are labeled as independently significant if their p-value <0.001 after adjusting for all other independently significant variables. Listed factors that were not independently significant had a p value of <0.001 after adjusting for age, race and study. Their tabulated chi-squared value were obtained after adjusting for the independently significant variables. ^‡^Change from minimum adult weight was only collected for participants in the observational study; ^§^The hazard ratio was computed for an increase in the variable of one standard deviation; ^∥^The hazard ratio is statistically significant at p<0.01 in this dataset.

**Table 5:**
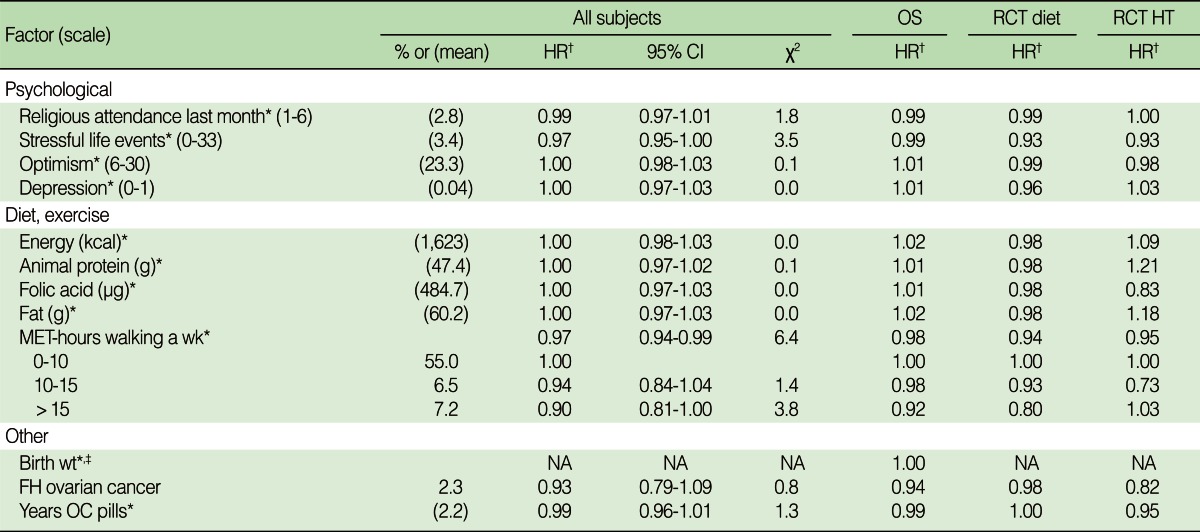
Variables of special interest that were not statistically significant at p<0.001 after adjusting for demographic factors

HR, hazard ratio; CI, confidence interval; OS, observational study; RCT, randomized controlled trial; RCT HT, combination of two RCTs; Wt, weight; FH, family history; MET, metabolic equivalent; OC, oral contraceptives. ^*^The hazard ratio was computed for an increase in the variable of one standard deviation; ^†^Hazard ratios were computed for an increase of 1 unit of the ordinal variable or comparison to the reference category of that variable. This reference category has a hazard ratio of 1.00. Hazard ratios were adjusted for all factors independently significant at p<0.001; ^‡^Only collected for patients in the observational study.
